# Continuous Spinal Anesthesia in a High‐Risk Patient With Pulmonary Hypertension Undergoing Hip Fracture Repair: A Case Report

**DOI:** 10.1155/cria/5262622

**Published:** 2026-09-02

**Authors:** Jaber El Kaissi, Jaouad Laoutid

**Affiliations:** ^1^ Anesthesiology & Intensive Care Department, Moulay Ismail Military Hospital, Meknes, Morocco; ^2^ Faculty of Medicine, Sidi Mohammed Ben Abdellah University, Fes, Morocco, usmba.ac.ma

**Keywords:** continuous spinal anesthesia, hip fracture surgery, pulmonary hypertension, regional anesthesia, right ventricular dysfunction

## Abstract

This report describes the successful use of continuous spinal anesthesia (CSA) in a 70‐year‐old woman with severe pulmonary hypertension (PH), right ventricular (RV) dysfunction, and obstructive sleep apnea (OSA) undergoing hip fracture repair. The perioperative challenge was to manage a patient at high risk of hemodynamic decompensation and severe gas exchange impairment. CSA allowed preservation of spontaneous ventilation and incremental titration of local anesthetic, while hemodynamic stability was maintained with early norepinephrine support. This case illustrates the importance of individualized, pathophysiology‐based anesthetic planning in high‐risk cardiopulmonary patients.

## 1. Introduction

Pulmonary hypertension (PH) and right ventricular (RV) dysfunction represent high‐risk comorbidities in perioperative care. These patients are particularly vulnerable to changes in preload, afterload, and pulmonary vascular resistance (PVR), especially during anesthesia. When associated with obstructive sleep apnea (OSA), the risk of perioperative decompensation is even higher. General anesthesia (GA) and mechanical ventilation may worsen this profile by increasing PVR and impairing RV function.

We report the use of continuous spinal anesthesia (CSA) in a patient with multiple cardiopulmonary risk factors. This approach avoided intubation and allowed precise control of sympathetic block, illustrating how incrementally titrated CSA may be incorporated into an individualized anesthetic strategy in selected high‐risk patients.

## 2. Case Presentation

A 70‐year‐old woman presented for hip fracture repair after a domestic fall. Her history included severe OSA requiring BiPAP, Group 1 PH (PAP 50 mmHg), and RV dysfunction. She also had systemic hypertension and obesity (BMI 33 kg/m^2^) and was ASA IV. Previous anesthetic history was unremarkable.

Initial arterial blood gas revealed pH = 6.99, PaCO_2_ = 60 mmHg, and PaO_2_ = 75 mmHg, indicating uncompensated hypercapnic respiratory failure.

The patient underwent full preoperative evaluation including echocardiography, ABG, and cardiopulmonary assessment. Principal concerns were RV decompensation, PH exacerbation, and ventilatory failure. Optimization included maintaining BiPAP preoperatively, optimizing intravascular volume, and planning advanced monitoring. Our patient was not under PH‐targeted therapies, but she was under ACE inhibitors and diuretics. We decided to continue both medications.

After multidisciplinary evaluation, CSA was selected as the anesthetic strategy. The anesthetic risks, benefits, and available alternatives were explained to the patient and her family before surgery, and informed consent was obtained for the anesthetic plan and surgical procedure.

Standard ASA monitoring was supplemented with invasive arterial pressure (left radial) and central venous access (right IJV). Both were performed under local anesthesia. No sedative medication was administered during placement of the arterial line or central venous catheter, during spinal catheter insertion, or at any time throughout the surgical procedure.

After skin preparation and draping, CSA was performed at the L2–L3 level using a PAUJUNK IntraLong kit with Sprotte needle. The spinal catheter was inserted with the patient in the sitting position, with careful assistance to minimize pain, respiratory compromise, and abrupt hemodynamic changes. A pericapsular nerve group (PENG) block was considered to facilitate positioning and provide perioperative analgesia. However, because the patient tolerated the sitting position without significant pain or respiratory compromise, the block was not performed. A skin incision was needed due to the softness of the introducer. A 3‐cm catheter was advanced into the intrathecal space.

CSA was administered using 0.5% hyperbaric bupivacaine. The protocol involved an initial intrathecal dose of 2.5 mg. After 15 min, a second 2.5 mg was administered to reach a T10 sensory level. Forty minutes into the procedure, the patient began to report mild discomfort, prompting an additional 2.5 mg dose. A final 2.5 mg top‐up was given at the conclusion of surgery, bringing the total dose to 10 mg. All injections were given via the intrathecal catheter over the course of the 90‐min surgery.

The surgical procedure was performed in the supine position. Positioning was carried out gradually and carefully, with attention to patient comfort. Verbal reassurance was maintained throughout the procedure.

Hemodynamics were maintained with norepinephrine infusion. The infusion was prepared before neuraxial block placement and initiated early after incremental intrathecal dosing when a downward trend in arterial pressure was observed. The infusion was titrated between 0.03 and 0.05 μg/kg/min, and it was discontinued before the end of surgery.

Cement was used during the orthopedic procedure, with no cement‐related hemodynamic or respiratory incident during cementation or in the postoperative period. Estimated intraoperative blood loss was approximately 200 mL, and no blood transfusion was required.

The patient remained awake and spontaneously breathing with oxygen via nasal cannula throughout the 90‐min procedure. Mild hypotension was promptly managed with norepinephrine, with no episodes of hypoxia, bradycardia, altered mental status, or cement‐related hemodynamic disturbance.

Postoperatively, BiPAP was resumed, and the patient was transferred to a step‐down unit. Analgesia was provided using a multimodal opioid‐sparing regimen. No respiratory deterioration, hemodynamic instability, arrhythmia, neurological complication, or need for rescue analgesia occurred. The spinal catheter was removed without incident, and the patient was discharged on postoperative day four in stable clinical condition.

## 3. Discussion

PH is a severe perioperative risk factor, particularly when associated with RV dysfunction. The RV is physiologically not designed to cope with high afterload. It is thin‐walled and highly sensitive to changes in PVR. In patients with PH, any increase in PVR or drop in systemic perfusion can lead to RV failure, decreased cardiac output, hypotension, and ultimately cardiovascular collapse [1].

Multiple perioperative triggers may worsen PH and compromise RV function: Positive pressure ventilation increases intrathoracic pressure and PVR, reducing venous return. Hypoxia, hypercapnia, acidosis, and pain are potent pulmonary vasoconstrictors. Anesthetic induction with agents causing vasodilation or myocardial depression can precipitate acute decompensation. Even minor hemodynamic shifts can have dramatic consequences in these fragile patients [2].

In this context, GA carries considerable risks. Induction may cause sudden vasodilation and hypotension. Tracheal intubation and mechanical ventilation can increase PVR and impair RV preload, triggering acute right heart failure. Moreover, volatile agents may depress myocardial contractility and interfere with pulmonary vascular tone [3].

Neuraxial anesthesia, when carefully titrated, may offer an alternative to GA in selected patients with PH [4]. CSA was considered particularly relevant in this case because it allowed incremental administration of small doses of local anesthetic while preserving spontaneous ventilation. Avoidance of tracheal intubation and positive‐pressure ventilation was considered desirable because increases in intrathoracic pressure, hypoxemia, hypercapnia, or hemodynamic instability could adversely affect PVR and RV function. Incremental intrathecal dosing also allowed gradual establishment of the sympathetic block and close titration of vasopressor support.

Several regional anesthetic techniques were considered. Single‐shot spinal anesthesia could have provided a reliable surgical block; however, even low‐dose spinal anesthesia may cause clinically relevant hypotension in elderly patients undergoing hip fracture repair [5]. This was an important concern because an abrupt decrease in systemic vascular resistance could compromise systemic pressure and RV coronary perfusion.

Epidural anesthesia was also considered because it permits incremental administration of local anesthetic and gradual extension of the neuraxial block. However, CSA was preferred because it was expected to provide a faster and more predictable surgical block using smaller doses of local anesthetic. In a randomized study of 30 patients older than 60 years, Reisli et al. compared CSA with continuous epidural anesthesia. Catheter placement was significantly faster with CSA than with continuous epidural anesthesia, requiring 3 ± 1.1 min versus 10 ± 2.2 min, respectively. The time required to reach the T10 sensory level and the maximum sensory level was also shorter with CSA, 18.0 ± 4.7 min versus 25.3 ± 7.0 min. After the initial dose, the block was inadequate for surgery in one patient in the CSA group and in three patients in the epidural group. Additional intraoperative local anesthetic was required in five patients receiving CSA and in 10 patients receiving continuous epidural anesthesia, and one patient in the epidural group required conversion to GA because of inadequate regional anesthesia. The decrease in mean arterial pressure was also significantly greater in the epidural group [6]. In our patient, a prolonged or incomplete block could have resulted in pain, sympathetic stimulation, repeated supplementation, or urgent conversion to GA. CSA was therefore considered the more predictable and titratable neuraxial technique for this particular clinical situation.

Combined spinal–epidural (CSE) anesthesia was another possible option because it permits extension of the neuraxial block through the epidural catheter. However, Imbelloni et al. reported that CSA required a lower total dose of bupivacaine than CSE, 8.5 ± 3.4 mg versus 18.7 ± 21.9 mg, and was associated with fewer episodes of arterial hypotension, occurring in 4 patients in the CSA group compared with 17 patients in the CSE group [7]. These findings supported the choice of a technique permitting incremental intrathecal dosing when close control of the sympathetic block was considered particularly important.

Peripheral nerve blocks were also considered. A combined psoas compartment–sciatic nerve block may limit neuraxial sympathetic blockade and, in a randomized study by Aksoy et al., was associated with greater early hemodynamic stability than CSA in elderly high‐risk patients [8]. Nevertheless, this approach requires two deep peripheral blocks, relatively large volumes of local anesthetic, and a longer procedural time, and it may result in incomplete surgical anesthesia requiring supplementation or conversion to GA. In this patient, CSA was considered more likely to provide a dense and predictable surgical block while maintaining spontaneous ventilation.

GA would have provided rapid airway control and predictable surgical conditions. However, induction‐related vasodilation, myocardial depression, tracheal intubation, and positive‐pressure ventilation could have caused significant hemodynamic and respiratory stress in a patient with PH, RV dysfunction, OSA, and hypercapnic respiratory failure. Nevertheless, GA remained the predefined rescue technique in the event of inadequate regional anesthesia, respiratory deterioration, or hemodynamic instability.

CSA was therefore selected as an individualized compromise between reliability of surgical anesthesia, incremental dosing, preservation of spontaneous ventilation, and hemodynamic titratability. Invasive arterial monitoring permitted early detection of changes in systemic pressure, while norepinephrine was prepared before neuraxial dosing and titrated according to the hemodynamic response. Although CSA was successfully used in this patient, the outcome of a single case cannot establish its superiority or general safety compared with epidural anesthesia, peripheral nerve blocks, or GA. Its use should be limited to carefully selected patients and settings with experienced operators, close monitoring, immediate vasopressor availability, and readiness to convert to GA.

The use of CSA in high‐risk patients undergoing hip fracture surgery is not novel in itself, and individualized neuraxial strategies have previously been reported. Aksoy et al. described CSA for hip fracture repair in a 55‐year‐old patient with severe left ventricular systolic dysfunction and an ejection fraction of 15%–20%. Their patient received intravenous midazolam, an initial intrathecal dose of 7.5 mg hyperbaric bupivacaine, and an additional 5 mg near the end of surgery. Hemodynamic stability was maintained without vasopressor therapy, and the spinal catheter was subsequently used for postoperative analgesia [9].

Khan et al. reported an 86‐year‐old patient with World Health Organization Group 2 PH, severe biventricular dysfunction, RV dilation and hypokinesia, and severe tricuspid regurgitation undergoing emergency hip fracture repair. Their anesthetic strategy consisted of intravenous fentanyl for positioning, a 10‐mg single‐shot spinal anesthetic, precautionary epidural catheter placement, invasive arterial monitoring, and low‐dose norepinephrine infusion; the epidural catheter was ultimately not used [10].

Although our case does not introduce a novel anesthetic technique, it adds to the existing literature by illustrating how CSA can be integrated into an individualized perioperative strategy for a patient with combined hemodynamic and respiratory vulnerability. Together with the previously reported cases, it emphasizes that anesthetic management should be guided by the dominant pathophysiological features of each patient rather than by the presumed superiority of a particular technique. Careful assessment of ventricular function, PVR, systemic perfusion, and ventilatory reserve is therefore essential when selecting the anesthetic approach.

As a single case, these findings cannot be generalized. However, CSA may be particularly useful in selected high‐risk patients with PH and limited cardiopulmonary reserve, when preservation of spontaneous ventilation and gradual sympathetic blockade are considered desirable.

### 3.1. Practical Learning Points

− In patients with PH and RV dysfunction, the anesthetic objective should not simply be avoidance of GA but preservation of RV perfusion and prevention of increases in PVR.
− When neuraxial anesthesia is selected, the speed and extent of sympathetic blockade are critical; CSA may be useful when a dense surgical block is required but abrupt sympathectomy is poorly tolerated.
− Vasopressor support should be anticipated and prepared before neuraxial dosing rather than used only as a late rescue measure after severe hypotension.
− In patients with OSA and hypercapnic respiratory failure, preservation of spontaneous ventilation and early postoperative resumption of noninvasive ventilation are central components of the anesthetic plan.

## Funding

This paper received no specific grant from any funding agency in the public, commercial, or not‐for‐profit sectors.

## Consent

Written informed consent was obtained from the patient for publication of this case report.

## Conflicts of Interest

Dr. Jaber El Kaissi declares that, after the initial submission of this manuscript and before revision, he was invited by PAJUNK to conduct live educational sessions during the World Congress of Anaesthesiologists in Marrakech. The other author declares no conflicts of interest.

## Supporting Information

Additional supporting information can be found online in the Supporting Information section.

## Supporting information


**Supporting Information** This case report was prepared in accordance with the CARE guidelines. A completed CARE checklist is provided as supporting information.

## Data Availability

The data that support the findings of this study are available from the corresponding author upon request.
